# An Intelligent Opportunistic Routing Algorithm for Wireless Sensor Networks and Its Application Towards e-Healthcare

**DOI:** 10.3390/s20143887

**Published:** 2020-07-13

**Authors:** Deep Kumar Bangotra, Yashwant Singh, Arvind Selwal, Nagesh Kumar, Pradeep Kumar Singh, Wei-Chiang Hong

**Affiliations:** 1 Department of Higher Education, J&K Govt, Jammu 180001, India; deepbangotra.ap@gmail.com; 2 Department of Computer Science and Information Technology, Central University of Jammu, J&K, Jammu 181001, India; yashwant.csit@cujammu.ac.in (Y.S.); arvind.cuj@gmail.com (A.S.); 3 Faculty of Engineering & Technology, Shoolini University of Biotechnology and Management Sciences, Solan 173211, India; engg.nagesh2@gmail.com; 4 ABES Engineering College, Ghaziabad, Uttar Pradesh 201009, India; 5 Department of Information Management, Oriental Institute of Technology, New Taipei 224, Taiwan

**Keywords:** wireless sensor networks (WSN), opportunistic routing (OR), naïve Bayes, relay node, energy efficiency, reliability

## Abstract

The lifetime of a node in wireless sensor networks (WSN) is directly responsible for the longevity of the wireless network. The routing of packets is the most energy-consuming activity for a sensor node. Thus, finding an energy-efficient routing strategy for transmission of packets becomes of utmost importance. The opportunistic routing (OR) protocol is one of the new routing protocol that promises reliability and energy efficiency during transmission of packets in wireless sensor networks (WSN). In this paper, we propose an intelligent opportunistic routing protocol (IOP) using a machine learning technique, to select a relay node from the list of potential forwarder nodes to achieve energy efficiency and reliability in the network. The proposed approach might have applications including e-healthcare services. As the proposed method might achieve reliability in the network because it can connect several healthcare network devices in a better way and good healthcare services might be offered. In addition to this, the proposed method saves energy, therefore, it helps the remote patient to connect with healthcare services for a longer duration with the integration of IoT services.

## 1. Introduction

WSN is a network of spatially dispersed tiny sensor nodes responsible for the collection of data from the physical environment. These sensor nodes are used for observing environmental parameters like pressure, moisture, temperature, wind speed, vibrations, heat, noise, etc., to transmit this acquired data to the sink or target node in the network. The recent improvements in the WSN technology have enabled the sensor nodes in the network, not only to capture real-time data but also to capture data in complicated real-life applications like a medical diagnosis of a heart patient, by implanting sensor nodes to record real-time data about the heart rate. These real-time applications demand real-time communication from the source node to the destination node. 

**Figure 1:**
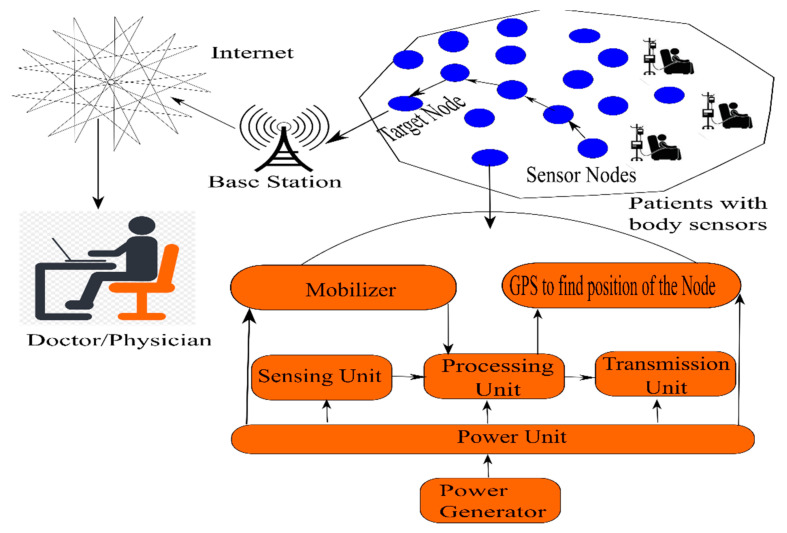
Sensor node architecture with application in e-healthcare.

The WSN is composed of tiny nodes called sensors, shown in Figure 1 [1]. These sensors form the basis of WSN because of their abilities to sense the environment, where they are deployed and also because of their ability to collaborate to form networks [2]. These sensor nodes are not of much use due to their limited possession of resources but once they create a network, they become very useful in the acquisition of environmental parameters, processing the acquired data and transmitting the processed data to a sensor node known as a sink in the WSN. A sensor node in WSN is capable of performing operations like sensing or acquisition, storage of acquired data, and transmission of data to different nodes in the network, so that the data reaches from the source node to the terminus node. It is important to understand that WSNs are subject to its own limitations and its implementation is a tough task, mainly due to their limited energy, poor communication range, very low storage capacity, and weak processing power [3]. Once the sensor nodes are deployed in the real environment, it is expected that they will remain operational for a stretched duration in unmanned sites. The whole scenarios demand efficient management of resources like processing, energy, storage, etc. The lifetime of the network is directly reliant on the amount of residual energy in the sensor motes, as it is nearly impossible to replace batteries in the sensor nodes. As sensor nodes are the nodes with a very limited amount of energy, it is very important to make optimum use of energy that is available with the sensor nodes. The maximum amount of energy is consumed during the communication operation. It is during communication when each node in the network behaves as a router and transmits the data packets from the source node to the next node, and then follows a route according to the protocol in use, so that the data packet reaches the destination. This task in the WSN demands maximum consumption of resources.

With the ever-increasing use of term green computing, the energy efficiency of WSN has seen a considerable rise. Recently, an approach for green computing towards IoT for energy efficiency has been proposed, which enhances the energy efficiency of WSN [4]. Different types of methods and techniques were proposed and developed in the past to address the issue of energy optimization in WSN. Another approach that regulates the challenge of energy optimization in sensor-enabled IoT with the use of quantum-based green computing, makes routing efficient and reliable [5]. The problem of energy efficiency during the routing of data packets from source to target in case of IoT-oriented WSN is significantly addressed by another network-based routing protocol known as GreeDi [6]. It is imperative to mention here that IoT is composed of energy-hungry sensor devices. The constraint of energy in sensor nodes has affected the transmission of data from one node to another and therefore, requires boundless methods, policies, and strategies to overcome this challenge [7]. 

The focus of this paper was to put forward an intelligent opportunistic routing protocol so that the consumption of resources particularly during communication could be optimized, because the alleyway taken to transmit a data packet from a source node to the target node is determined by the routing protocol. 

Routing is a complex task in WSN because it is different from designing a routing protocol in traditional networks. In WSN, the important concern is to create an energy-efficient routing strategy to route packet from source to destination, because the nodes in the WSN are always energy-constrained. The problem of energy consumption while routing is managed with the use of a special type of routing protocol known as the Opportunistic Routing Protocol. The opportunistic routing (OR) is also known as any path routing that has gained huge importance in the recent years of research in WSN [8]. This protocol exploits the basic feature of wireless networks, i.e., broadcast transmission of data. The earlier routing strategies consider this property of broadcasting as a disadvantage, as it induces interference. The focal notion behind OR is to take the benefit of spreading the behavior of the wireless networks such that broadcast from one node can be listened by numerous nodes. Rather than selecting the next forwarder node in advance, the OR chooses the next forwarder node robustly at the time of data transmission. It was shown that OR gives better performance results than traditional routing. In OR, the best opportunities are searched to transmit the data packets from source to destination [9]. The hop-by-hop communication pattern is used in the OR even when there is no source-to-destination linked route. The OR protocols proposed in recent times by different researchers are still belligerent with concerns pertaining to energy efficiency and the reliable delivery of data packets. 

The proposed OR routing protocol given in this paper was specifically meant for WSN, by taking into account the problems that surface during the selection of relay candidates and execution of coordination protocol. The proposed protocol intelligently selects the relay candidates from the forwarder list by using a machine learning technique to achieve energy efficiency. The potential relay node selection is a multi-class with multiple feature-based probabilistic problems, where the inherent selection of relay node is dependent upon each node’s characteristics. The selection of a node with various characteristics for a node is a supervised multiclass non-linearly separable problem. In this paper, the relay node selection algorithm is given using Naïve Baye’s machine learning model.

The organization of this paper is as follows. Section 2 presents the related work in the literature regarding OR and protocols. The various types of routing protocols are given in Section 3. Section 4 describes OR with examples, followed by the proposed intelligent OR algorithm for forwarder node selection in Section 5. Section 6 depicts the simulation results of the proposed protocol by showing latency, network lifetime, throughput, and energy efficiency. Section 7 presents a proposed framework for integration IoT with WSN for e-healthcare. This architecture can be useful in many e-healthcare applications. Section 8 presents the conclusion and future.

## 2. Related Work

Achieving reliable delivery of data and energy efficiency are two crucial tasks in WSNs. As the sensor nodes are mostly deployed in an unattended environment and the likelihood of any node going out of order is high, the maintenance and management of topology is a rigorous task. Therefore, the routing protocol should accommodate the dynamic nature of the WSNs. Opportunistic routing protocols developed in the recent past years provided trustworthy data delivery but they are still deficient in providing energy-efficient data transmission between the sensor nodes. Some latest research on OR, experimented by using the formerly suggested routing metrics and they concentrated on mutual cooperation among nodes.

GeRaF [10] (Geographic Random Forwarding) described a novel forwarding technique based on the geographical location of the nodes involved and random selection of the relaying node via contention among receivers. Exclusive Opportunistic multi-hop routing for wireless networks [11] (ExOR) is an integrated routing and MAC protocol for multi-hop wireless networks, in which the best of multiple receivers forwards each packet. This protocol is based on the Expected Transmission Count (ETX) metric. The ETX was measured by hop count from the source to the destination and the data packet traveled through the minimum number of hops. ExOR achieves higher throughput than traditional routing algorithms but it still has few limitations. ExOR contemplates the information accessible at the period of transmission only, and any unfitting information because of recent updates could worsen its performance and could lead to packet duplication. Other than this, there is another limitation with ExOR, as it always seeks coordination among nodes that causes overhead, in case of large networks. Minimum Transmission Scheme-Optimal Forwarder List Selection in Opportunistic Routing [12] (MTS) is another routing protocol that uses MTS instead of ETX as in ExOR. The MTS-based algorithm gives fewer transmissions as compared to ETX-based ExOR. Simple, practical, and effective opportunistic routing for short-haul multi-hop wireless networks [13]. In this protocol, the packet duplication rate was decreased. This is a simple algorithm and can be combined with other opportunistic routing algorithms. Spectrum Aware Opportunistic Routing [14] (SAOR) is another routing protocol for the cognitive radio network. It uses optimal link transmission (OLT) as a cost metric for positioning the nodes in the forwarder list. SAOR gives better QoS, reduced end-to-end delay, and improved throughput. Energy-Efficient Opportunistic Routing [15] (EEOR) calculates the cost for each node to transfer the data packets. The EEOR takes less time than ExOR for sending and receiving the data packets. Trusted opportunistic routing algorithm for Vanet [16] (TMCOR) gives a trust mechanism for opportunistic routing algorithm. It also defines the trade-off between the cost metric and the safety factor. A novel socially aware opportunistic routing algorithm in mobile social networks [17] considered three parameters, namely social profile matching, social connectivity matching, and social interaction. This gives a high probability of packet delivery and routing efficiency. ENSOR-opportunistic routing algorithm for relay node selection in WSNs is another algorithm where the concept of an energy-efficient node is implemented [18]. The packet delivery rate of ENSOR is better than GeRaF. Economy—a duplicate free [19] is the only OR protocol that uses token-based coordination. This algorithm ensures the absence of duplicate packet transmissions.

With the advent of the latest network technologies, the virtualization of networks along with its related resources has made networks more reliable and efficient. The virtual network functions are used to solve the problems related to service function chains in cloud-fog computing [20]. Further, IoT works with multiple network domains, and the possibility of compromising the security and confidentiality of data is always inevitable. Therefore, the use of virtual networks for service function chains in cloud-fog computing under multiple network domains, leads to saving network resources [21]. In recent times, the cloud of things (CoT) has gained immense popularity, due to its ability to offer an enormous amount of resources to wireless networks and heterogeneous mobile edge computing systems. The CoT makes the opportunistic decision-making during the online processing of tasks for load sharing, and makes the overall network reliable and efficient [22]. The cloud of things framework can significantly improve communication gaps between cloud resources and other mobile devices. In this paper, the author(s) proposed a methodology for offloading computation in mobile devices, which might reduce failure rates. This algorithm reduces failure rates by improving the control policy. In recent times, WSN used virtualization techniques to offer energy-efficient and fault-tolerant data communication to the immensely growing service domain for IoT [23]. With the application of WSN in e-healthcare, the wireless body area network (WBAN) gained a huge response in the healthcare domain. The WBAN is used to monitor patient data by using body sensors, and transmits the acquired data, based on the severity of the patients’ symptoms, by allocating a channel without contention or with contention [24].

EEOR [15] is an energy-efficient protocol that works on transmission power as a major parameter. This protocol discussed two cases that involved constant and dynamic power consumption models. These models are known as non-adjustable and adjustable power models. In the first model, the algorithm calculated the expected cost at each node and made a forwarder list on the source node based on this cost. The forwarder list was sorted in increasing order of expected cost and the first node on the list became the next-hop forwarder. As EEOR is an opportunistic routing protocol, broadcasting is utilized and the packets transmitted might be received by each node on the forwarder list. In this, the authors propose algorithms for fixed-power calculation, adjustable power calculation, and opportunistic power calculation. This algorithm was compared with ExOR [11] by simulation in the TOSSIM simulator. The results showed that EEOR always calculated the end-to-end cost based on links from the source to destination. EEOR followed distance vector routing for storing the routing information inside each sensor node. The expected energy consumption cost was updated inside each node, after each round of packet transmission. Data delivery was guaranteed in this protocol. Additionally, according to the simulation results, packet duplication was significantly decreased.

The MDOR [25,26] protocol worked on the distance between the source to relay nodes. In this, the authors proposed an algorithm that calculated the distance to each neighbor from the source node and found out the average distance node. The average distance node was used by the source as a next-hop forwarder. The authors also stated that, to increase the speed and reliability of transmission, the strength of the signal was very important. The signal power depended on the distance between the sender and receiver. If a node sent a packet to the nearest node, then it might take more hops and this would decrease the lifetime of the network. Another problem addressed in this protocol was to reduce energy consumption at each node through the dynamic energy consumption model. This model consumed energy according to the packet size and transmitted the packet by amplifying it according to the distance between the source and the relay nodes. MDOR always chose the middle position node to optimize energy consumption in amplifying the packets. The MDOR simulation results showed that the energy consumption was optimized and it was suitable for certain applications of WSN like environment monitoring, forest fire detection, etc.

Opportunistic routing introduced the concept of reducing the number of retransmissions to save energy and taking advantage of the broadcasting nature of the wireless networks. With broadcasting, the routing protocol could discover as many paths in the network as possible. The data transmission would take place on any of these paths. If a particular path failed, the transmission could be completed by using some other path, using the forwarder list that had the nodes with the same data packet.

## 3. Routing Protocols in WSN

The protocols that were responsible for data transmission in WSN were broadly ordered into two sets [2], namely, (i) old-fashioned routing, and (ii) opportunistic routing. In the traditional routing, also known as old-fashioned routing techniques, the focus was on finding the route with a minimum number of intermediate nodes from the source to the destination, without taking into consideration some of the important factors like throughput, quality of links, reliability, etc. A small comparison [27] of the routing categories is shown in Table 1.

As it is clear from the literature that energy consumption of a sensor node had a considerable impact on the lifetime and quality of the wireless sensor network, therefore, it becomes vital to design energy-efficient opportunistic routing protocols to maximize the overall lifetime of the network and also to enhance the quality of the sensor network. There are few methods in the literature listed below that might be useful to save the life of the sensor network.

- Scheduling of duty cycle
- Energy-efficient medium access control (EE-MAC)
- Energy-efficient routing
- Node replacements (not possible in unattended environments)
- Energy harvesting
- Energy replenishment
- Energy balance

Of the above-mentioned methods for energy saving, energy-efficient routing is the most central method for the vitality of the WSN. As this method involved the transmission of signals, i.e., receiving and sending, it took about 66.66 percent of the total energy of the network [28]. Therefore, it became relevant that an opportunistic routing protocol that enhanced the vitality of the sensor network might be designed for enhancing the overall life span of the sensor network.

## 4. Proposed Algorithm

### 4.1. Opportunistic Routing (OR)

OR broadcasts a data packet to a set of relay candidates that is overheard by the neighboring nodes, whereas in traditional routing a node is (pre)-selected for each transmission. Then, relay candidates that are part of the forwarders list and who have successfully acknowledged the data packet, run a protocol called coordination protocol between themselves, to choose the best relay candidate to onward the data packet. In other words, OR is abstractly comprised of these three steps:

Step 1: Broadcast a data packet to the relay candidates (this will prepare the forwarder list).
Step 2: Select the best relay by using a coordination protocol among the nodes in the forwarder list.
Step 3: Forward the data packet to the selected relay node.

**Figure 2:**
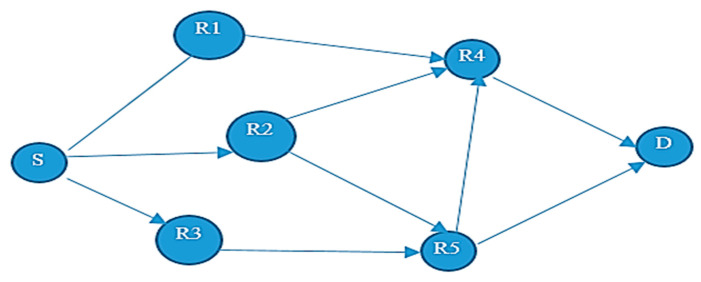
Depiction of opportunistic routing in wireless sensor networks (WSN).

Considering an example shown in Figure 2, where the source node S sends a packet to the destination node D, through nodes R1, R2, R3, R4, and R5. First, S broadcasts a packet. The relay nodes R1, R2, and R3 might become the forwarder nodes. Further, if R2 is chosen as a potential forwarder, then R4 and R5 might become relay nodes. Similarly, if R5 is the forwarder node, then it forwards the data packets to the destination node D.

Opportunistic Routing Derived the Following Rewards:

- *The escalation in reliability*. By using this routing strategy, the reliability of WSN increased significantly, as this protocol transmitted the data packet through any possible link rather than any pre-decided link. Therefore, this routing protocol provided additional links that could act as back up links and thus reduced the chances of transmission failure.
- *The escalation in transmission range*. With this routing protocol, the broadcast nature of the wireless medium provided an upsurge in the transmission range, as all links irrespective of their location and quality of data packets were received. Hence, the data transmission could reach the farthest relay node successfully.

### 4.2. Proposed Machine Learning-Based Opportunistic Routing (OR) Protocol

#### 4.2.1. Preliminaries

In WSN, the sensor nodes could be deployed in two ways, randomly or manually. Most applications require the random deployment of nodes in the area under consideration. Initially, each node is loaded with the same amount of battery power. As soon as the network starts functioning, the nodes start consuming energy. To make the network energy efficient, the protocol used for transmitting data packets must consume less battery power and the calculation of the energy consumption network model and energy model should be formulated. In the upcoming subsection, these two models are discussed and these are depicted as assumptions, to carry out smooth working of the protocol.

#### 4.2.2. Network Model

The N sensors are distributed in a square area of size 500 ∗ 500 square meters. This network formed a graph G = (N, M), with the following possessions:

- N = {N_1_, N_2_, …, N_n_} is the set of vertices representing sensor nodes.
- M is considered to be a set of edges representing the node-to-node links.
- The neighboring list NBL(N*_i_*) consists of nodes that are in the direct link to the N_i_.
- The data traffic is assumed to be traveling from the sensor nodes toward the base station.
- If a packet delivery is successful, then the acknowledgment (ACK) for the same is considered to travel the same path back to the source.

#### 4.2.3. Energy Model

The lifespan of a WSN depends on the endurance of each node, while performing network operations. The sensor nodes rely on the battery life to perform network operations. The energy cost model considered here is the first-order energy model for WSN [25]. Various terms used in Equations (1)–(3) are defined in Table 2.

Energy Consumed in the transmission of *n*-bit packet up to *l* distance:

(1) $$E_{trs}\left(n,l\right)=n*E_{radio}+n*E_{amplification}*l^{2}$$

Energy Consumed in the transmission of n-bit packet:

(2) $$E_{rsx}\left(n\right)=n*E_{radio}$$

Energy Consumed in receiving and sending acknowledgements:

(3) $$E_{ACK}\left(n,l\right)=E_{T_{elec}}\left(n,l\right)+E_{R_{elec}}\left(n\right)\;=2nE_{elect}+n*E_{amplification}*l^{2},\;if\;l<l_{0}\;=2nE_{elect}+n*E_{amplification}*l^{4},\;if\;l\geq l_{0}$$

#### 4.2.4. Sensor Node’s Board Operation

Sensor Board—full operation, radio board—full operation, CPU board—sleep, wakeup for creating messages only.

The proposed protocol uses these assumptions as preliminaries. A new algorithm is proposed in the next section, for solving the issue of energy efficiency and the reliability of opportunistic routing in WSN.

## 5. Intelligent Opportunistic Protocol (IOP)

Let there be N nodes in the WSN, where each node has K neighbors, i.e., N_1_, N_2_, …, N_K_ and each neighbor nodes are represented by X_1_, X_2_, …, X_n_ attributes. In this case, the number of neighbors (k) might vary for the different nodes at a particular instance. Additionally, it was assumed that the wireless sensor network is spread over an area of 500 × 500 square meters.

Let us assume that a node A ∈ N and had neighbors as NA_1_, NA_2_, …, NA_K_, with respective features like Node Id, Location, PRR (Packet Reception Ratio), Residual Energy (RE) of nodes, and Distance (D), which are represented by X_1_, X_2_, …, X_n_, respectively. The goal was to intelligently find a potential relay node A, say AR, such that AR ∈ {NA_1_, NA_2_, …, NA_K_}. In the proposed machine learning-based protocol for the selection of potential forwarder, the packet reception ratio, distance, and outstanding energy of node was taken into consideration. The Packet Reception Ratio (PRR) [29] is also sometimes referred to as PSR (packet success ratio). The PSR was computed as the ratio of the successfully received packets to the sent packets. A similar metric to the PRR was the PER (packet error ratio), which could be computed as (1—PRR). A node loses a particular amount of energy during transmission and reception of packets. Accordingly, the residual energy in a node gets decreased [30]. The distance (D) was the distance between the source node and the respective distance of each sensor node in the forwarder list. The potential relay node selection was multi-class, with multiple features-based probabilistic problems, where the inherent selection of the relay node was dependent upon each node feature. The underlying probabilistic-based relay node selection problem could be addressed intelligently by building a machine learning model. The selection of a node with ‘n’ characteristics for a given node ‘A’ could be considered a supervised multiclass non-linearly separable problem.

In this algorithm, the Naïve Baye’s classifier was used to find the probability of node A to reach one of its neighbors, i.e., {N_1_, N_2_, …, N_K_}. We computed the probability, P(N_1_, N_2_, …, N_K_|A). The node with maximum probability, i.e., P(N_1_, N_2_, …, N_K_|A) was selected. The probability P of selecting an individual relay node of the given node A could be computed individually for each node, as shown respectively for each node in Equation (4).

(4) $$P\left(NA_{1}|A\right)=P\left(X_{1}\right)*P\left(X_{2}\right)\ldots \ldots \ldots \ldots \ldots .P\left(X_{n}\right)\;P\left(NA_{2}|A\right)=P\left(X_{1}\right)*P\left(X_{2}\right)\ldots \ldots \ldots \ldots \ldots .P\left(X_{n}\right)\;\ldots \ldots \ldots \ldots \ldots \ldots \ldots \ldots \ldots \ldots \ldots \ldots \ldots \ldots \ldots \ldots \;\ldots \ldots \ldots \ldots \ldots \ldots \ldots \ldots \ldots \ldots \ldots \ldots \ldots \ldots \ldots \ldots \;P\left(NA_{K}|A\right)=P\left(X_{1}\right)*P\left(X_{2}\right)\ldots \ldots \ldots \ldots \ldots .P\left(X_{n}\right)$$

where P(NA_K_|A) denotes the probability of node A to node K. Furthermore, the probability computation of node A to NA_1_ is such that NA_1_ is represented by the corresponding characteristics X_1_, X_2_, …, Xn, which means to find the probability to select the relay node NA_1_, given that feature X_1_, NA_1_ given that feature X_2_, NA_1_ given that feature X_3_, and so on. The individual probability of relay node selection, given that the node characteristics might be computed by using Naïve Bayes conditional probability, is shown in Equation (5).

(5) $$P(A|X_{i})=\frac{P(X_{i}|A)*P\left(A\right)}{P\left(X_{i}\right)}$$

where *i* = 1, 2, 3, …, n and P(X*i*|A) is called likelihood, P(A) is called the prior probability of the event, and P(X*i*) is the prior probability of the consequence. The underlying problem is to find the relay node A that has the maximum probability, as shown in Equation (6).

(6) $$P\left(A|NA_{1},NA_{2},\ldots \ldots \ldots NA_{K}\right)=max((P(NA_{1}|A_{1})|(P\left(NA_{2}|A_{1}\right)\ldots \ldots \ldots (P(NA_{K}|A_{1})$$

Table 3a–x represent the neighbor sets {NA_1_, NA_2_, …, NA_K_} along with their feature attributes as {X_1_, X_2_, X_3_, …, X_n_} of Node A. The working of IOP is comprised of two phases, i.e., Phase I (Forwarder_Set_Selection) and Phase II (Forwarder_Node_Selection). In Phase I, the authors used Algorithm 1 for the forwarder set selection. In this step, the information collection task was initiated after the nodes were randomly deployed in the area of interest, with specific dimensions. The beginning of the phase started with a broadcast of “Hello Packet” which contained the address and the location of the sending node. If any node received this packet, it sent an acknowledgment to the source and was added to the neighbor list.

This process was repeated again and again, but not more than the threshold, to calculate the PRR of each node and the neighbor list was formed using this procedure repeatedly. From the neighbor list and the value of PRR, the forwarder set was extracted. The pre-requisite for the working of the second phase was the output of the first phase. The forwarder set generated from Algorithm 1 was the set of all nodes that had the potential to forward the data packets. However, all nodes in the set could not be picked for transmission, as this would lead to duplication of packets in the network. To tackle this situation, only one node from the forwarder list should be selected to transmit the packet to the next-hop toward the destination. This was accomplished using Algorithm 2, which took a forwarder node list as input and selected a single node as a forwarder. Algorithm 2 used a machine-learning technique called naïve Baye’s Classifier, to select the forwarder node intelligently.

The proposed method of relay node selection using IOP could be understood by considering an example of WSN shown in Figure 2 and using the naïve Baye’s algorithm on the generic data available in Table 4, to find the optimal path in terms of energy efficiency and reliability from source node S to destination node D. Therefore, by using the proposed naïve Baye’s classifier method, the probability of selection of a relay node R1, R2, or R3 from source node S was denoted by P(R1, R2, R3|S), which could be calculated using Equation (7).
**Algorithm 1.** Forwarder_Set_Selection (S = Source, D = Destination)**//When any source node S want to send a packet towards D, it will execute this algorithm//****Notations**NBL: Neighbor listREP_Count: Count of replies received by the source nodeSID: Identity of the source nodeRES: Energy residual of the nodePRR: Packet Reception RatioFL(S): Forwarder List of source node S**Input**  Source Node S, SID, RES, and Location corresponding to the node S**Process**  **START**  Let NBL (S) be the neighbor list of node S  Let REP_Count (node): = 0  For count: = 1 to 5 repeat  Broadcast “Hello_Packet” as {SID, Coordinates (x, y), RES} from S;  IF (reply == True and node !Ɛ NBL(S))  Add the replying node to the neighbor list NBL(S) with following values updated    {Node_ID, Location, Energy};  Else IF (reply == True and node Ɛ NBL(S))  REP_Count (node) = REP_Count (node) + 1;  Else  count = count + 1;  endIF  count = count + 1;  endFor  For each node in NBL (S) repeat  Calculate PRR (node);  IF PRR (node) >= 0.2  Add node to forwarder set (FL(S));  endIF  endFor  Forwarder Set FL(S) is formed;  **END****Output:** Creation of forwarder list consisting of neighbor nodes of the source node S.

(7) $$P\left(R1|S\right)=P\left(R1_{PRR}|S\right)*P\left(R1_{RE}|S\right)*P\left(R1_{D}|S\right)$$

Using Equation (5), we can compute:

where,

$$P\left(R1_{PRR}|S\right)=\frac{P\left(S|R1_{PRR}\right)*P\left(R1_{PRR}\right)}{P\left(S\right)}$$

$$P\left(R1_{RE}|S\right)=\frac{P\left(S|R1_{RE}\right)*P\left(R1_{RE}\right)}{P\left(S\right)}$$

$$P\left(R1_{D}|S\right)=\frac{P\left(S|R1_{D}\right)*P\left(R1_{D}\right)}{P\left(S\right)}$$

Putting the values in the above equations from Table 4, we can compute

(8) $$P\left(R1_{PRR}|S\right)=\frac{P\left(S|R1_{PRR}\right)*P\left(R1_{PRR}\right)}{P\left(S\right)}=\frac{\left(\frac{0.79}{64.09}\right)*\left(\frac{0.79}{2.58}\right)}{\frac{1}{3}}=\frac{\left(0.012\right)*\left(0.30\right)}{0.33}=0.010$$

**Algorithm 2.** Forwarder_Node_Selection (S = Source, D = Destination)//when any node S wants to send a packet toward D and it has already constructed a forwarder list, it will follow this algorithm.
**Notations**
FL(S):Forwarder List of source node SRi:ith node in the forwarder listPRR:Packet Reception Ratio)RE:Residual EnergyD:DistanceP:ProbabilityArrProb[Ri]:Array to store probability of a given nodePmax:Maximum probabilityD:Destination/Target

**Input**
  R: R is set of nodes in the forwarder list (FL) i.e., R = {R1, R2, …, Rn}**Process**  **START**Let R1, R2, …, Rn are the nodes in the FL of source node S. Each node Ri∈ FL(S) where i = 1, 2, …, nDeclare three float variables X_1_, X_2_, and X_3_ to represent the properties of Ri, i.e., PRR (Packet Reception Ratio), RE (Residual Energy), and D (distance), respectively.For each node Ri∈ FL(S) repeatCompute P(Ri|S)//Probability of selection of Ri given S, i.e.,P_k_ = P(R_i_|S) for i = 1, 2 …, n and Assign k←iCompute the probability of P(R_i_|S) by computing the probability of each parameter separately, given S. $P_{k}=P\left(Ri|S\right)=P\left(Ri_{X1}|S\right)*P\left(Ri_{X2}|S\right)*P\left(Ri_{X3}|S\right)$
where,
$$P\left(Ri_{X1}|S\right)=\frac{P\left(S|Ri_{X1}\right)*P\left(Ri_{X1}\right)}{P\left(S\right)}$$
$$P\left(Ri_{X2}|S\right)=\frac{P\left(S|Ri_{X2}\right)*P\left(Ri_{X2}\right)}{P\left(S\right)}$$
$P\left(Ri_{X3}|S\right)=\frac{P\left(S|Ri_{X3}\right)*P\left(Ri_{X3}\right)}{P\left(S\right)}$Make an unsorted array of probability values of n nodes, i.e., R1, R2, …, Rn from step 6.For i = 1 to n and k = i, ArrProb[Ri]←P_k_//To find the node with maximum probability.Select the first node of the array ArrProb[0] as the node with maximum value Pmax i.e., Pmax←ArrProb[0]Go through the rest of the elements of the array, i.e., from the 2nd element to the last (n − 1)element, for i = 1 to n − 1.For i = 1 to n − 1, if any value in the array ArrProb[i] is found to be greater than the current value Pmax. i.e.,if ArrProb[i] > Pmax then, Pmax← ArrProb[i]When the end of the array is reached, then the current value of the Pmax is the greatest value in the array, Pmax←ArrProb[i].The node Ri with Pmax value is selected as a relay node from the forwarder list, asthe node with the highest probability. The node with the next highest probability acts as a relay node in case the first selected relay node fails to broadcast.Broadcast transmission of the data packet as {Ri, coordinates, data}Destination node D is reached, if Yes, go to step 15. Else, apply Algorithm 1 on RiS←Ri and go to step 2.**END****Output:** A potential forwarder node is selected from the list of forwarder nodes.

(9) $$P\left(R1_{RE}|S\right)=\frac{P\left(S|R1_{RE}\right)*P\left(R1_{RE}\right)}{P\left(S\right)}=\frac{\left(\frac{49.3}{64.09}\right)*\left(\frac{49.3}{147.4}\right)}{\frac{1}{3}}=\frac{\left(0.76*0.33\right)}{0.33}=0.76$$

(10) $$P\left(R1_{D}|S\right)=\frac{P\left(S|R1_{D}\right)*P\left(R1_{D}\right)}{P\left(S\right)}=\frac{\left(\frac{14}{64.09}\right)*\left(\frac{14}{39}\right)}{\frac{1}{3}}=\frac{0.21*0.35}{0.33}=0.22$$

Using the above results, we can compute the probability of P(R1|S) by multiplying $P\left(R1_{PRR}|S\right)*P\left(R1_{RE}|S\right)*P\left(R1_{D}|S\right)$, i.e.,

(11) $$P\left(R1_{\mathrm{PRR}}|S\right)*P\left(R1_{\mathrm{RE}}|S\right)*P\left(R1_{D}|S\right)=0.010*0.76*0.22=0.001$$

Similarly, we can compute the probability of P(R2|S) by multiplying $P\left(R2_{PRR}|S\right)*P\left(R2_{RE}|S\right)*P\left(R2_{D}|S\right)$, i.e.,

(12) $$P\left(R2_{PRR}|S\right)*P\left(R2_{RE}|S\right)*P\left(R2_{D}|S\right)\;P(R2|S)=P\left(R2_{PRR}|S\right)*P\left(R2_{RE}|S\right)*P\left(R2_{D}|S\right)\;P\left(R2_{PRR}|S\right)=\frac{P\left(S|R2_{PRR}\right)*P\left(R2_{PRR}\right)}{P\left(S\right)}$$

(13) $$P\left(R2_{RE}|S\right)=\frac{P\left(S|R2_{RE}\right)*P\left(R2_{RE}\right)}{P\left(S\right)}$$

(14) $$P\left(R2_{D}|S\right)=\frac{P\left(S|R2_{D}\right)*P\left(R2_{D}\right)}{P\left(S\right)}$$

Again, inputting the values in the above Equations (12)–(14) from the table given below, we can compute:

(15) $$P\left(R2_{PRR}|S\right)=\frac{P\left(S|R2_{PRR}\right)*P\left(R2_{PRR}\right)}{P\left(S\right)}=\frac{\left(\left(\frac{0.87}{63.97}\right)*\left(\frac{0.87}{2.58}\right)\right)}{\frac{1}{3}}=\frac{0.013*0.33}{0.33}=0.013$$

(16) $$P\left(R2_{RE}|S\right)=\frac{P\left(S|R2_{RE}\right)*P\left(R2_{RE}\right)}{P\left(S\right)}=\frac{\left(\left(\frac{48.1}{63.97}\right)*\left(\frac{48.1}{147.4}\right)\right)}{\frac{1}{3}}=\frac{0.75*0.32}{0.33}=0.72$$

(17) $$P\left(R2_{D}|S\right)=\frac{P\left(S|R2_{D}\right)*P\left(R2_{D}\right)}{P\left(S\right)}=\frac{\left(\frac{15}{63.97}\right)*\left(\frac{15}{39}\right)}{\frac{1}{3}}=\frac{0.23*0.38}{0.33}=0.26$$

Therefore,

(18) $$P\left(R2|S\right)=P\left(R2_{\mathrm{PRR}}|S\right)*P\left(R2_{\mathrm{RE}}|S\right)*P\left(R2_{D}|S\right)=0.013*0.72*0.26=0.002$$

In the same manner, we can compute P(R3|S) by multiplying $P\left(R3_{PRR}|S\right)*P\left(R3_{RE}|S\right)*P\left(R3_{D}|S\right)$, i.e.,

(19) $$P\left(R3_{PRR}|S\right)*P\left(R3_{RE}|S\right)*P\left(R3_{D}|S\right)P\left(R3_{PRR}|S\right)=\frac{P\left(S|R3_{PRR}\right)*P\left(R3_{PRR}\right)}{P\left(S\right)}$$

(20) $$P\left(R3_{RE}|S\right)=\frac{P\left(S|R3_{RE}\right)*P\left(R3_{RE}\right)}{P\left(S\right)}$$

(21) $$P\left(R3_{D}|S\right)=\frac{P\left(S|R3_{D}\right)*P\left(R3_{D}\right)}{P\left(S\right)}$$

Again, putting the values in the above Equations (19)–(21) from the table given below, we can compute:

(22) $$P\left(R3_{PRR}|S\right)=\frac{P\left(S|R3_{PRR}\right)*P\left(R3_{PRR}\right)}{P\left(S\right)}=\frac{\left(\frac{0.92}{60.92}\right)*\left(\frac{0.92}{2.58}\right)}{\frac{1}{3}}=\frac{0.015*0.35}{0.33}=0.015$$

(23) $$P\left(R3_{RE}|S\right)=\frac{P\left(S|R3_{RE}\right)*P\left(R3_{RE}\right)}{P\left(S\right)}=\frac{\left(\frac{50}{60.92}\right)*\left(\frac{50}{147.4}\right)}{\frac{1}{3}}=\frac{0.82*0.33}{0.33}=0.82$$

(24) $$P\left(R3_{D}|S\right)=\frac{P\left(S|R3_{D}\right)*P\left(R3_{D}\right)}{P\left(S\right)}=\frac{\left(\frac{10}{60.92}\right)*\left(\frac{10}{39}\right)}{\frac{1}{3}}=\frac{0.16*0.25}{0.33}=0.12$$

Therefore,

(25) $$P(R3|S)=P\left(R3_{PRR}|S\right)*P\left(R3_{RE}|S\right)*P\left(R3_{D}|S\right)=0.015*0.82*0.12=0.001$$

Finally using the proposed method of relay node selection using naïve Baye’s algorithm, we could compute probability P(R1,R2,R3|S), using Equation (26).

(26) P(R1,R2,R3|S)=max(P(R1|S),P(R2|S),P(R3|S)=max(0.001,0.002,0.001)

Thus, node R2 would be selected as the relay node in the forwarder list of R1, R2, and R3 for source node S. Similarly, the process was followed again for the neighbors of S, which consequently would check the neighbors of R1, R2, and R3. The Table 5, Table 6 and Table 7 describe the features of neighboring nodes of R1, R2, and R3, respectively.

**Figure 3:**
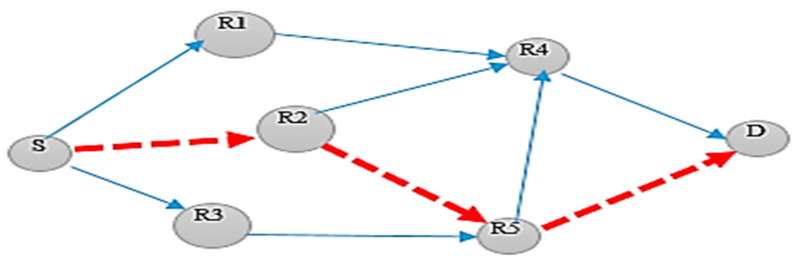
Illustration of the final route selection using IOP.

After the execution of Phase I and Phase II on the above said example, the final route was intelligently selected for the onward transmission of the data packet from source node S to destination node D, using the naïve Baye’s algorithm shown in Figure 3.

Figure 3 gives the details about the route selected using the IOP. The source node S broadcasts the data packet among its neighboring nodes, using Algorithm 1 to create a forwarders list. The node R1, R2, and R3 in the figure, were selected as the nodes in the forwarders list.

These were the potential nodes that would be used for the selection of a potential forwarder node. Here, R2 was selected as the potential node using Algorithm 2. The same procedure was adopted again and until the data reached its final destination. The final route was selected intelligently using IOP is S→R2→R5→D.

## 6. Simulation and Results

With the end goal of examination and comparison of the proposed OR protocol, the simulation was performed in MATLAB. The simulation used the environment provided by the MATLAB to simulate the computer networks and other networks. MATLAB provides a good scenario to design a network of sensor nodes and also to define a sensor node and its characteristics. The simulation results were compared with the results of the EEOR [25] and the MDOR [26] protocols. Table 8 below shows the parameter setting of the network.

### 6.1. Deployment of Sensor Nodes

**Figure 4:**
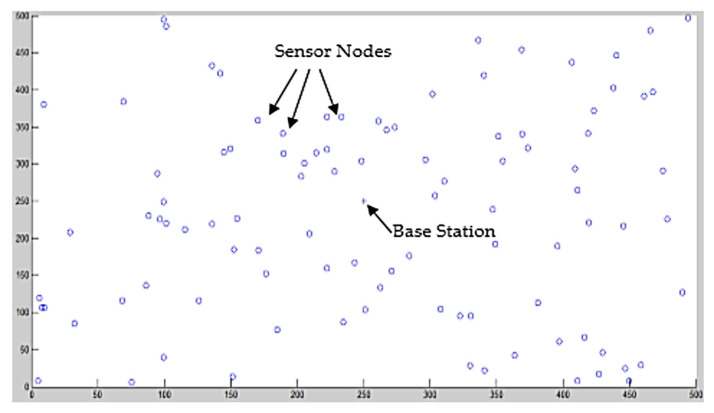
Random deployment of sensor nodes in 500 × 500 m^2^ area.

The motes are haphazardly deployed in 500 × 500 m field. The nodes are deployed in such a way that these can approximately cover the whole application area. The base station position is 250 × 250 m in the field. The field area was considered a physical world environment. The proposed OR protocol started working immediately after the deployment process was complete. Figure 4 below represents the unplanned deployment of the nodes in the area of consideration.

### 6.2. Energy Efficiency

**Figure 5:**
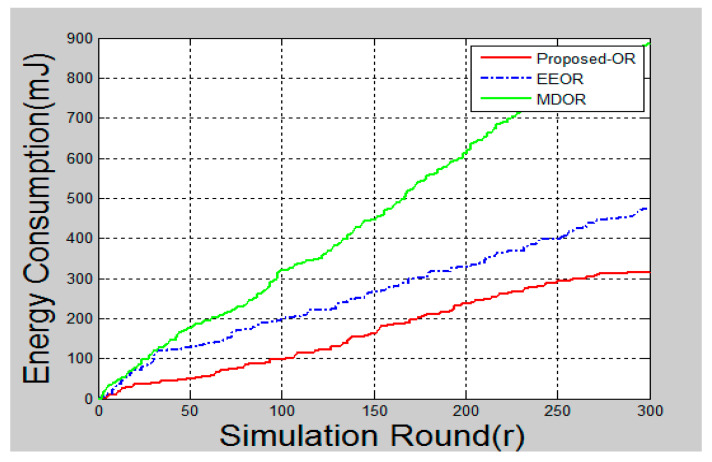
Total energy consumption.

Energy efficiency was the main objective of the proposed algorithm. It could be calculated as the overall energy consumption in the network for the accomplishment of diverse network operations. In MATLAB, the simulation worked based on simulation rounds. The simulation round was termed as packets transmission from a single source to a single destination. In MATLAB, when the simulation starts, a random source is chosen to start transmission and this node makes a forwarder list and starts executing the proposed protocol. One round of simulation represents successful or unsuccessful transmissions of packets from one source in the network. For each round, different source and relay nodes are selected. This process continues until at least one node is out of its energy. The energy efficiency was calculated as the total energy consumption after each round in the network. After the operation of the network starts, the sensor’s energy starts decaying. This energy reduction was due to network operations like setting up the network, transmission, reception, and acknowledging the data packets, processing of data, and sensing of data. As the nodes decayed, their energy consumption kept increasing per round, as can be seen in Figure 5 below. It can be seen in the figure that energy consumption for the proposed OR protocol was less, as compared to the other two algorithms. This was because the proposed OR protocol distributed energy consumption equally to all nodes, so that every node could survive up to their maximum lifetime. Hence, the proposed OR protocol was more energy-efficient than MDOR and EEOR.

### 6.3. Latency (End-to-End Delay)

Latency can be measured as the time elapsed between sending the packet and receiving the same at the base station. This is also called as end-to-end delay for the packets to be reached at the destination.

**Figure 6:**
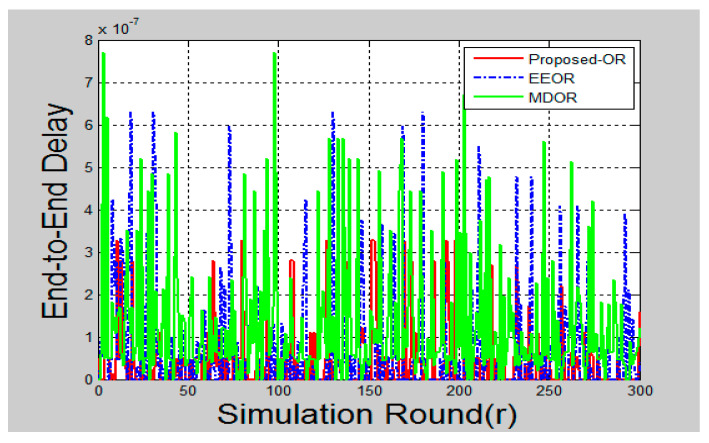
End-to-end delay.

The communication in wireless sensor networks is always from source nodes to the sink station. In the random deployment of nodes, some nodes are able to communicate directly with the base station. While some nodes follow multi-hop communication, i.e., source nodes have to go through relay nodes to forward the data packet toward the base station. Hence, in some cases, the network delay can be very low and in some cases, it can be high. Hence in Figure 6, the values of end-to-end delay after each communication in each round are plotted. It can be seen that the proposed OR protocol has a good latency, as compared to the other two protocols.

### 6.4. Throughput

The throughput of a network can be measured in different ways. Throughput is calculated as the average number of packets received successfully at the base station per second in each round.

**Figure 7:**
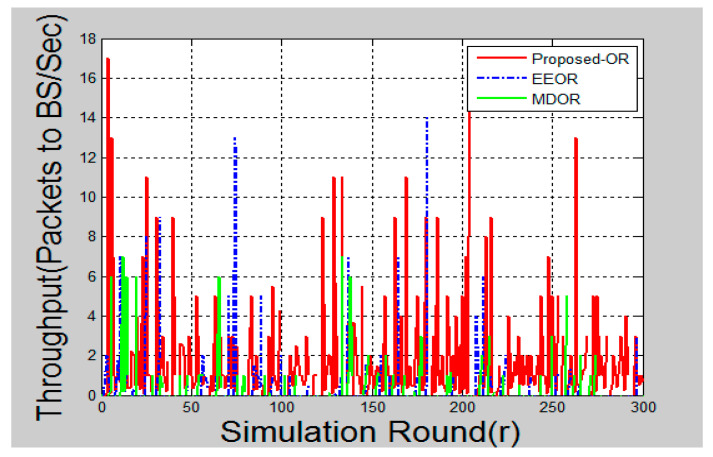
Packet to Base Station per Second.

Figure 7 represents the throughput for each round. The proposed OR protocol has good throughput, as compared to the other two. As the proposed OR protocol is efficient in energy consumption, the sensor nodes are able to survive and communicate for a long time in the network. As long as the communication goes on, the base station would continue to receive the packets.

### 6.5. Network Lifetime

**Figure 8:**
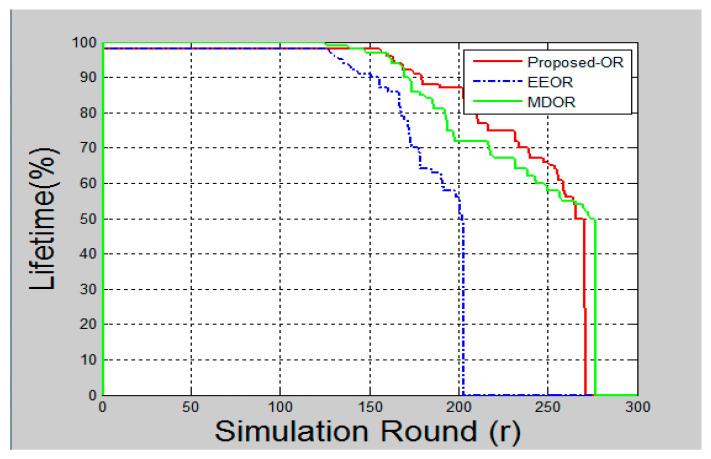
Network lifetime.

Network lifetime for wireless sensor networks is dependent upon the energy consumption in the network. When the energy of the network is 100 percent, the network lifetime would also be 100 percent. However, as the nodes start operating in the network, the network lifespan would start to reduce. Figure 8 represents the percentage of lifetime remaining after each round of simulation. Proposed OR protocol has a good network lifetime due to the lower energy consumption in the network.

### 6.6. Packet Loss

**Figure 9:**
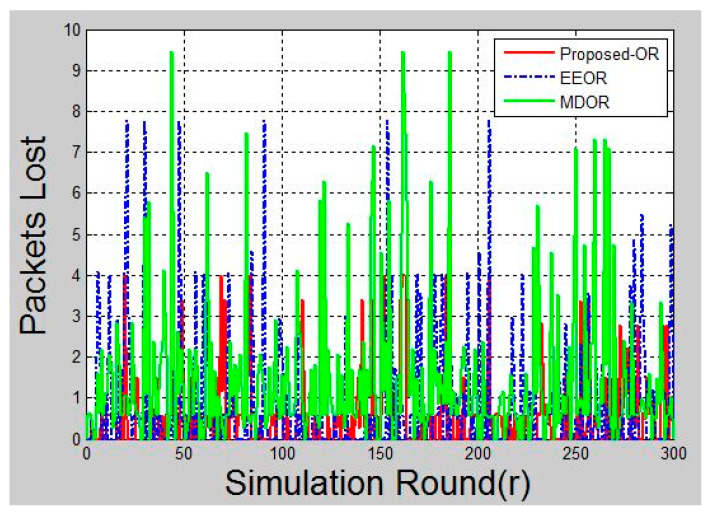
Packet loss during simulation.

The packet loss is referred to as the number of packets that are not received at the destination. To calculate the number of packets lost during each round of the simulation, packet sequence numbers are used. Whenever a source tries to send packets to a destination, it inserts a sequence number. Later, on packet reception, these packet sequence numbers are checked for continuity. If a certain sequence number is missing then it is referred to as packet loss. Packet loss recorded per round of simulation and presented in Figure 9. It can be depicted from the figure that packet loss for the proposed protocol is less, as compared to EEOR and MDOR. This is because the forwarder node selection algorithm runs on each relay and source node. This algorithm calculates the probability of successful transmission through a neighbor node. This also increases the reliability of the protocol and provides accurate transmissions.

### 6.7. Discussions

A significant improvement could be seen in the graphs after the simulation is complete. Figure 5 shows the total energy consumption after each round of packet transmission is complete. Here, the round was termed as packet transmissions in between single source and destination. MDOR showed the highest energy consumption, followed by EEOR and the proposed protocol. This was because MDOR wasted more energy in the initial setup. However, the dynamic energy consumption considerations led the network to survive for a long time, as shown in Figure 8. In the case of EEOR in Figure 5, it consumed lesser energy in transmission and the initial setup for opportunistic selection of relay nodes was based on the power level. However, when it comes to lifetime, EEOR failed to perform better, as it considered the network to be dead when any one of the nodes ran out of its energy. EEOR chose one node as a source and continued transmissions opportunistically, which resulted in a significant reduction in the power level of a single node. The proposed protocol gave the best results, as in each round, the source node was based on the intelligent model to change the next-hop relay node. Figure 6 presents the average end-to-end delay per round, generated by the simulation, and the proposed protocol worked significantly better as the next-hop selection was based on an intelligent algorithm. The proposed algorithm helped to significantly reduce average end-to-end delays. Figure 7 and Figure 9 showed the reliability and availability performances of all protocols, including the proposed protocol that showed significantly better performance. This meant that the proposed protocol was a new generation protocol that has potential in many applications of WSN.

## 7. Proposed Framework

**Figure 10:**
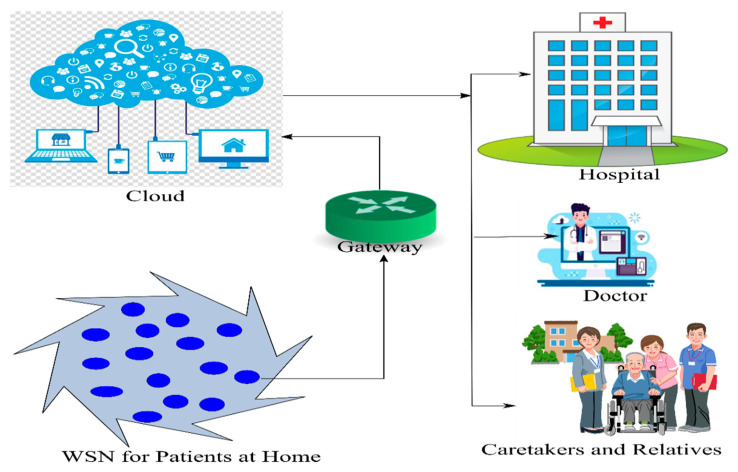
The proposed framework for integration of IoT with WSN for e-healthcare.

In recent years, WSN saw its applications growing exponentially with the integration of IoT. This gave a new purpose to the overall utility of data acquisition and transmission. With the integration of WSN with IoT, the IoT is making a big impact in diverse areas of life, i.e., e-healthcare, smart farming, traffic monitoring and regulation, weather forecast, automobiles, smart city, etc. All these applications are hugely dependent on the availability of real-time accurate data. Healthcare with IoT is one such area that involves critical decision making [31,32,33]. The proposed approach makes use of intelligent routing and, therefore, would help in making reliable and accurate delivery of data to the integrated healthcare infrastructure, for proper care of the patients. The proposed framework for e-healthcare is shown in Figure 10. As the proposed algorithm saves energy, the healthcare devices that are sensor-enabled can work for longer duration, and easy deployment and data analysis is possible due to IoT integration [34,35,36,37,38]. According to the proposed architecture, there can be any different kind of sensor nodes, such as smart wearables, sensors collecting health data like temperature, heartbeat, number of steps taken every day, sleep patterns, etc. These factors have a correlation with different existing diseases. The best part of the integration of IoT and WSN is that, with the help of sensors, data are collected and the same is stored in the cloud due to IoT integration. Once the health data is stored in the cloud, this cloud is a health-record cloud that belongs to a specific hospital or a public domain cloud. These cloud data can be accessed by healthcare professionals in a different way, to analyze the data and also provide feedback to a specific patient and group of patients.

In the recent epidemic of COVID-19, telemedicine had become one of the most popular uses of this platform. Doctors also started e-consulation to the patients and getting access to their health records, using the smart wearables of patients. Sill, there are many challenges, and lot of improvements are required. The proposed work add towards better energy efficiency of sensors, so that they can work for longer durations. Thereafter these sensor data can be integrated using IoT and cloud, as per the proposed approach shown in Figure 10.

## 8. Conclusion and Future Work

In this paper, we proposed a new routing protocol (IOP) for intelligently selecting the potential relay node using naïve Baye’s classifier to achieve energy efficiency and reliability among sensor nodes. Residual energy and distance were used to find the probability of a node to become a next-hop forwarder. Simulation results showed that the proposed IOP improved the network lifetime, stability, and throughput of the sensor networks. The proposed protocol ensured that nodes that are far away from the base station become relay nodes, only when they have sufficient energy for performing this duty. Additionally, a node in the middle of the source and destination has the highest probability to become a forwarder in a round. The simulation result showed that the proposed OR scheme was better than MDOR and EEOR in energy efficiency and network lifetime. Future work will examine the possibility of ensuring secure data transmission intelligently over the network.

## Figures and Tables

**Table 1:** Comparative study between opportunistic routing and traditional routing.

Routing Feature	Opportunistic Routing (OR)	Traditional Routing (TR)
Transmission type	Broadcast	Unicast
Data packets overheard	Yes	No
Relay selection	Dynamic	Fixed
Number of candidates	Multiple	Relay alone (Single)

**Table 2:** Description of various terms used in the equations.

Parameter	Description
$E_{trs:Ni\to fwd}$	Transmission vitality ingestion for node N_i_
$E_{rsx:Ni}$	Receiving vitality ingestion for node N_i_
$E_{ret:Ni\to fwd}$	Retransmission vitality ingestion for node N_i_
$E_{ACK:Ni\to source}$	Vitality spent by node N_i_ in transmitting and receiving acknowledgments
$RE_{Ni}$	The residual vitality of node N_i_
$E_{Trans}$	Transmission vitality cost of the radio board of a sensor
$E_{Receive}$	Reception vitality cost of the radio board of a sensor
$E_{Forward}$	Combined vitality cost of radio board of a sensor for communication of a data packets

**Table 3:** Description of node features for different neighbor nodes of A.

(**a**)				
**Neighbours of A1**	**Attributes**			
	**X_1_**	**X_2_**	**…**	**X_n_**
N_A_^1^				
N_A_^2^				
…….				
N_A_^K^				
(**b**)				
**Neighbours of A2**	**Attributes**			
	**X_1_**	**X_2_**	**…**	**X_n_**
N_A_^1^				
N_A_^2^				
…….				
N_A_^K^				
…				
(**x**)				
**Neighbours of A_n_**	**Attributes**			
	**X_1_**	**X_2_**	**…**	**X_n_**
N_A_^1^				
N_A_^2^				
…….				
N_A_^K^				

**Table 4:** Description of neighbors of source node S along with its features.

	Attributes					
Neighbors of Source Node S	Node_Id (NID)	Location (LOC)	Packet Reception Ratio (PRR)	Residual Energy (RE) In Joules	Distance (D) (in meters)	
R1	S0001	(20,90)	0.79	49.3	14	64.09
R2	S0002	(25,110)	0.87	48.1	15	63.97
R3	S0003	(60,150)	0.92	50	10	60.92

**Table 5:** Description of neighbors R1 along with its features.

Neighbors of Source Node R1	Attributes				
	Node_Id	Location	PRR	Residual Energy (J)	Distance (m)
R4	R10001	(35,100)	0.4	0.2	12

**Table 6:** Description of neighbors of R2 along with its features.

Neighbors of Source Node R2	Attributes				
	Node_Id	Location	PRR	Residual Energy (J)	Distance (m)
R4	R10004	(35,100)	0.4	0.2	12
R5	R20005	(49,79)	0.6	0.7	11

**Table 7:** Description of neighbors of R3 along with its features.

Neighbors of Source Node R3	Attributes				
	Node_Id	Location	PRR	Residual Energy (J)	Distance (m)
R5	R20005	(49,79)	0.6	0.7	11
D					

**Table 8:** Simulation Parameters.

Simulation Parameter	Value
Area	500 × 500 m
Number of Nodes	100
Initial Energy of Each node	0.5 Joule
Electronic Energy (Eelec)	50 nJ (50 × 0.000000001 Joule)
Amplification Energy (Eamp)	100 pJ (10 × 0.000000000001 Joule)
Packet Size	50 bits
Number of Simulation Rounds	100
Threshold Energy Eth	0.2 Joules
